# Patient safety culture in a university hospital

**DOI:** 10.1590/1518-8345.2257.3014

**Published:** 2018-08-09

**Authors:** Taís Freire Galvão, Marcélia Célia Couteiro Lopes, Carmen Conceição Carrilho Oliva, Maria Elizete de Almeida Araújo, Marcus Tolentino Silva

**Affiliations:** 1 PhD, Adjunct Professor, Faculdade de Ciências Farmacêuticas, Universidade Estadual de Campinas, Campinas, SP, Brazil.; 2 MSc, Pharmacist, Hospital Universitário Getúlio Vargas, Universidade Federal do Amazonas, Manaus, AM, Brazil.; 3 MSc, Pharmacist, Unidade Básica de Saúde Leonor de Freitas, Secretaria Municipal de Saúde, Manaus, AM, Brazil.; 4 PhD, Pharmacist, Hospital Universitário Getúlio Vargas, Universidade Federal do Amazonas, Manaus, AM, Brazil.; 5 PhD, Adjunct Professor, Faculdade de Medicina, Universidade Federal do Amazonas, Manaus, AM, Brazil. Adjunct Professor, Universidade de Sorocaba, Sorocaba, SP, Brazil.; Universidade de Sorocaba, Universidade de Sorocaba, Sorocaba, SP, Brazil

**Keywords:** Patient Safety, Organizational Culture, Hospitals, Delivery of Health Care, Health Personnel, Surveys and Questionnaires

## Abstract

**Objective:**

to assess patient safety culture in a university hospital.

**Method:**

cross-sectional study with data collection through the Hospital Survey on Patient Safety Culture applied in electronic device. A total of 381 employees were interviewed, corresponding to 46% of the sum of eligible professionals. Data were analyzed descriptively. the Cronbach’s alpha was used to calculate the frequency and reliability.

**Results:**

most were women (73%) from the nursing area (50%) and with direct contact with patients (82%). The composites related to “teamwork within units” (58%, α=0.68), “organizational learning - continuous improvement” (58%, α=0.63), “supervisor/manager expectations and actions promoting patient safety” (56%, α=0.73) had higher positive responses. Nine composites had low positive responses, with emphasis on “nonpunitive response to error” (18%, α=0.40). Only the item “in this unit, people treat each other with respect” had positive response above 70%. The patient safety assessment in the work unit was positive for 36% of employees, however only 22% reported events in past year.

**Conclusion:**

the findings revealed weaknesses in the safety culture at the hospital, with emphasis on culpability.

## Introduction

Patient safety culture corresponds to values and behaviors of members in an institution and collectively represents the degree of institutional commitment with the safety of its processes^1^. This construct reflects intangible aspects of health care, influenced exceedingly by the leadership, supervision and feedback to professionals^2^. Caregivers recognize to be inserted into an institution in which to follow the procedures is important. Therefore, they mark out their actions by performing the good practices of the area and providing information for its continuous improvement^3^.

Institutions with patient safety potentially provide safe care of better quality to their patients. The best scores on dimensions regarding safety culture were related to the lower incidence of surgical site infection in hospital^4^, reduction of injuries, critical adverse events and risk-adjusted mortality^5^. In risk-adjusted morbidity analyses of the patients and characteristics of the hospital, however, the positive responses of safety culture were not related to mortality in patients with acute myocardial infarction^6^, nor was affected after reduction of catheter-associated infections^7^.

The safety culture in healthcare environments is typically assessed by quantitative surveys based on individual items and combination of composites^1^. In Brazil, the National Patient Safety Program (*Programa Nacional de Segurança do Paciente*), established by the Ordinance 529/2013 of the Brazilian Ministry of Health, has safety culture as implementation strategy. The evaluation of patient safety culture is the first step to find the aspects that require improvement in this process.

In the Brazilian context, some initiatives to measure and evaluate safety culture in institutions have been registered^8^ ^-^ ^11^, revealing weaknesses in different aspects. There still prevails the perception that failures in patient safety point to individual responsibilities and, consequently, punitive actions for the professional. This posture prevents the establishment of the improvements required. In the Northern Region of Brazil, which is historically less developed and with lower supply of health professionals and services^12^, this scenario is possibly more prevalent. This region of the country lacks investigations on safety culture. The objective of this research was to assess the patient safety culture in a university hospital from Manaus, Amazonas.

## Methods

Cross-sectional study developed in the Getulio Vargas University Hospital, in Manaus, Amazonas. It is a teaching hospital of the Federal University of Amazonas, managed by the Brazilian Company of Hospital Services (*Empresa Brasileira de Serviços Hospitalares*) and contracted by the Brazilian Unified Health System. The research was conducted from June to September 2015.

Healthcare and administrative employees (including public servants, temporary employees or professionals of the multi-professional and medical residency program) working at least for three months in the institution were elected. Employees that were separated, on leave, or worked outside the main building of the hospital were ineligible.

Participants were selected by convenience sampling. A schedule to visit all sectors in the three shifts and weekends was prepared in the period of the research. A total of 381 employees were interviewed, corresponding to 46% of the sum of eligible professionals. Before the beginning of the interviews, the hospital commissioner communicated the managers about the research and encouraged the participation of employees. To inform the objectives and convoke the participants, advertisements about the research were posted in the murals of the hospital.

The primary outcome was defined as the proportion of positive responses in each composite of the Hospital Survey on Patient Safety Culture (HSOPS). Demographic (sex, age, educational level) and professional (work unit, staff position or function, how long he/she has been working in the hospital, weekly workload) variables were collected for sample characterization.

The HSOPS was translated, transculturally adapted and validated for use in the Brazilian context^13^ ^-^ ^14^. The survey consisted of 42 questions distributed in 12 composites and three levels: (i) work unit (supervisor/manager expectations and actions promoting patient safety; organizational learning - continuous improvement; staffing; communication openness; feedback and communication about error; nonpunitive response to error; and teamwork within units, (ii) hospital organization (management support for patient safety; teamwork across units; and handoffs and transitions) and (iii) results (patient safety grade; and frequency of events reported). The two questions of result (perception of patient safety and number of safety events reported in the last 12 months) were evaluated separately, without constituting composites.

The responses of HSOPS were codified by the Likert scale of five points (agreement: strongly disagree, disagree, neither, agree, strongly agree; frequency: never, rarely, sometimes, most of the time, always). The results were evaluated based on the performance of each item and composite. The items and composites with 75% of positive responses were considered strong and the ones less than 50% were considered weak^15^.

The Portuguese version of the HSOPS was loaded in electronic questionnaire in the KoboToolbox software and made available in tablets of the Samsung Tab-3 SM-T110. The questions were sequentially disposed and configured with mandatory responses in each question to avoid data loss. The research team tested the electronic survey questionnaire to verify the understanding of questions and adequacy of the survey to the interface adopted.

In these rounds, the need to improve the writing of three questions of the HSOPS was observed, as stated in a previous analysis^16^. The term “event reports” in questions C1 and G1 was replaced by “notifications”, term consolidated in Brazilian health services. Question A5 was written as “sometimes, the best patient care is not provided due to the excessive workload” instead of “staff (regardless of employment relationship) in this unit work longer hours than is best for patient care”^16^.

Undergraduate students, pharmacy and medicine residents and employees from the sector of Health and Patient Safety Surveillance of Brazil were trained to conduct the interviews, which occurred in the sector and working hours of the employees.

After the participant signed the informed consent form, the interviewer explained how to answer the questionnaire in the tablet. The device was delivered and the interviewer stood available for answering potential questions.

We aimed at minimizing the risk of selection bias by previous communicating the occurrence of the survey and sending motivational messages to encourage the participation of employees in the research. Refusals were registered to the assessment of the response rate of the survey.

The choice of using questionnaires in tablets, which were filled out by the professional, was due to the goal of ensuring the confidentiality and avoiding embarrassment of the participant in informing data of personal (feelings, expectations) and professional nature (insecure behaviors, conceptions on the institution and management). Such cautions aimed at minimizing risk of measurement bias.

Because it is a descriptive research, the calculation of sample size was dismissed. The maximum number of professionals available in the study period and in all shifts of work was invited.

The variables collected were statistically described. The questions of the HSOPS were grouped in the 12 composites, and the ones with negative responses were reversed. The proportion of positive responses to each item was calculated: the numerator was the total of positive responses and the denominator was the total of respondents.

The reliability of the composites was calculated using the Cronbach’s alpha. Values ≥0.6 were considered of good reliability. The Stata 14.2 software was used for all calculations. Missing data were excluded from the analysis, without imputation.

The project was approved by the Research Ethics Committee of the Universidade Federal do Amazonas, through the opinion 1,082,410 from 05/27/2015, certificate of presentation for ethical consideration (CAAE) 44286115.0.0000.5020 of the *Plataforma Brasil*.

## Results

**Figure 1:**
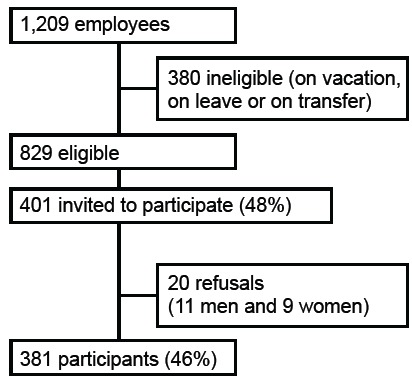
Selection process of the survey participants at the university hospital, Manaus, AM, 2015

A total of 401 employees were invited to participate in the study and 381 accepted (response rate: 95%), which represented 46% of eligible employees (Figure 1).

Sociodemographic characteristics shown in Table 1 demonstrate that most of the respondents were women with mean age of 39±11 years. More than 80% had direct contact with patients and 50% had graduate studies. Half of respondents was from the nursing body, among technicians (35%) and nurses (15%) and have been worked there for a year (50%). Most had weekly workload between 20 and 39 hours (66%). 

**Table 1:** Characteristics of professionals interviewed at the university hospital, Manaus, AM, Brazil, 2015 (n=381).

Characteristic		n (%)
	Female	278 (73)
	Age (mean±SD*)	38.6±11.0
	Direct contact with patients	310 (81)
Educational level		
	High school^†^	107 (28)
	Complete higher education	83 (22)
	Graduate studies	191 (50)
Work unit		
	Diverse	120 (31)
	Surgical	85 (22)
	Clinic	67 (18)
	Diagnostic and therapeutic support^‡^	65 (17)
	Intensive care	45 (12)
Position or function in the hospital		
	Nurse technician	132 (35)
	Nurse	58 (15)
	Another higher-level professional^§^	58 (16)
	Physician	40 (10)
	Administrative	37
	Technician^||^	28 (7)
	Other	28 (7)
Time working in the hospital (years)^¶^		
	<1	137 (50)
	1 to 10	112 (40)
	>11	127 (46)
Weekly workload (hours)		
	less than 20 to 39	252 (66)
	40 to 59	82 (22)
	> 60	47 (12)

* standard deviation

† includes 4 people with some high school

‡ rehabilitation, pharmacy, laboratory, radiology

§ physical therapist, nutritionist, pharmacist, biologist, social worker, psychologist, dentist

|| electrocardiography, laboratory, radiology, pharmacy

¶ 5 interviews missing this information

According to Table 2, the composites with greater proportion of positive responses were: teamwork within units (58%); organizational learning - continuous improvement (58%); and supervisor/manager expectations and actions promoting patient safety (56%). The others had less positive responses than 50%, and the composite “nonpunitive response to error” had the lowest rate (18%).

The HSOPS had good reliability using the Cronbach’s alpha (0.63−0.88), except for the composites of “overall perceptions of patient safety” (0.48), “staffing” (0.42) and “nonpunitive response to error” (0.40).

**Table 2:** Proportion of positive responses and reliability using the Cronbach’s alpha (α) of each composite of the Hospital Survey on Patient Safety Culture instrument at the university hospital, Manaus, AM, Brazil, 2015 (n=381)

Patient safety culture composite	%	α
Teamwork within units	58	0.68
Organizational learning - continuous improvement	58	0.63
Supervisor/manager expectations and actions promoting patient safety	56	0.73
Frequency of events reported	44	0.88
Communication openness	41	0.64
Feedback and communication about error	38	0.75
Teamwork across units	37	0.66
Handoffs and transitions	36	0.71
Management support for patient safety	35	0.78
Overall perception of patient safety	33	0.48
Staffing	33	0.42
Nonpunitive response to error	18	0.40

The majority of items (31/42) had negative responses, and only the item A4 - “in this unit, people treat each other with respect” had more than 70% of positive responses (data not presented).

Patient safety culture assessment in the work unit was positive for 36% of employees, according Table 3. Of these, the majority filled out no reports in the last 12 months (78%) and 2% filled out six reports or more.

**Table 3:** Quality of patient safety in the unit and number of reports filled out in the last 12 months at the university hospital, Manaus, AM, Brazil, 2015 (n=376)

Variables		N* (%)
Patient safety grade		
	Excellent	22 (6)
	Very good	113 (30)
	Acceptable	192 (51)
	Poor	35 (9)
	Failing	14 (4)
Number of event reports filled out in the last 12 months		
	No reports	294 (53)
	1 to 2	53 (30)
	3 to 5	22 (13)
	6 or more	7 (4)

* 5 interviews missing these variables

## Discussion

The safety culture measured by the HSOPS showed weaknesses for the university hospital assessed. Only three composites had positive responses above 50% and none represented strengths (above 75%) in patient safety culture.

The instrument used had good reliability using the Cronbach’s alpha in two thirds of the composites. The strategy used to improve the understanding of some questions, as pointed by other researchers^16^, increased the reliability of the composites in relation to validation^14^. Another strategy would be the exclusion of low-performance questions^14^, however the instrument would have less items than the HSOPS originally developed. A new version of the HSOPS was validated for the Brazilian context and developed in an interface of electronic application^17^. The reliability of the instrument was high (α=0.92), possibly avoiding the interpretation limitations of the version applied in this investigation^14^.

The composite with lowest proportion of positive responses was the “nonpunitive response to errors”, which also had the lowest reliability. In addition to this composite having a problematic aspect in institutions - the culpability culture -, it consisted of only negative questions, which required higher attention on interpretation and had less reliability in questionnaires^18^. Analyses of psychometric properties of HSOPS point to possible weaknesses in measuring the patient safety culture^19^. Composites with lower scores may reflect the writing of items and not necessarily the weaknesses in safety culture.

The result found in the composite “nonpunitive response to error” resembles studies carried out in intensive care in Brazil, in which this composite had the lowest proportion between composites of patient safety culture (14% to 29%)^8^ ^,^ ^20^ ^-^ ^21^. These lower positive responses were also observed in a systematic review with meta-analysis, in which seven of 11 studies included showed the lowest frequencies in the composite^22^.

Another factor that limits the results is the selection process by convenience of respondents, which decreases the representativeness of the hospital staff. The HSOPS ignores the recommendations on the sampling process - thus, the questionnaire can be forwarded by e-mail and only the respondents are analyzed^15^. We know that recruitment of participants influences the results, especially in internet surveys^23^. On the other hand, almost half of all employees eligible to the survey were interviewed and included in this study.

Our findings proportionally had more positive responses than a study carried out in Southern Region of Brazil in 2016 with 59 participants of the health team of an intensive care unit, whose variation was from 14% to 47% of positive responses^21^. On the other hand, we had less positive responses than study carried out in 2014 in a teaching hospital of São Paulo with 88 health professionals, in which the safety culture reached proportions between 29% to 75% (nonpunitive response to error and supervisor/manager expectations and actions promoting patient safety, respectively)^8^.

Composites with better scores (organizational learning - continuous improvement, teamwork within units and supervisor/manager expectations and actions promoting patient safety) were similar to the strengths observed in Saudi studies, but had modest positive responses given other international studies^20^ ^,^ ^24^ ^-^ ^26^.

Most respondents reported no adverse events in the past year. If on the one hand there is recognition of error and the importance of communicating it, on the other hand there is omission of it due to absence of communication^27^. Previous studies had better results, with proportions of reports between 22% to 53%^8^ ^,^ ^20^ ^-^ ^22^ ^,^ ^25^. National estimates indicate incidence of 5% of preventable adverse events during hospitalization^28^. The systemic approach to error, as opposed to the culpability, is strategic to improve the healthcare processes, covering the human nature involved in the processes and the complexity of health activities^29^. Unsafe procedures must be redesigned and monitored to avoid the occurrence of the error, which results from latent and active faults in the system and not from an isolated individual.

Our findings result from the interviews with almost half of the total of eligible employees based on a valid instrument to measure the patient safety culture in a university hospital. The findings possibly resemble other contexts of the Brazilian Unified Health System, which suffer with the underfunding. We highlight that this research establishes the first effort in measuring the patient safety culture in the Northern Region of Brazil.

## Conclusion

The patient safety culture in the university hospital was evaluated as still fragile. To invest in systematic approach to errors, professional team and management is a priority to strengthen the patient safety at hospital. The implementation and assessment of improvements in care, associated with the systematic measurement of the safety culture are strategies to increase the patient safety in hospital.
